# Effects of High Hydrostatic Pressure on Expression Profiles of *In Vitro* Produced Vitrified Bovine Blastocysts

**DOI:** 10.1038/srep21215

**Published:** 2016-02-17

**Authors:** Zongliang Jiang, Patrick Harrington, Ming Zhang, Sadie L. Marjani, Joonghoon Park, Lynn Kuo, Csaba Pribenszky, Xiuchun (Cindy) Tian

**Affiliations:** 1 Center for Regenerative Biology, Department of Animal Science, University of Connecticcut, Storrs, Connecticut, 06269, USA; 2 Department of Statistics, University of Connecticut, Storrs, Connecticut, 06269, USA; 3 State Key Laboratory for Conservation and Utilization of Subtropical Agro-bioresources, Animal Reproduction Institute, Guangxi University, Nanning, Guangxi, China, 510005; 4 Department of Biology, Central Connecticut State University, New Britain, Connecticut 06050, USA; 5 Laboratory of Assisted Reproduction, Department of Herd Health, University of Veterinary Science, Istvan u. 2. 1078 Budapest, Hungary

## Abstract

High hydrostatic pressure (HHP) has been used to pre-condition embryos before essential, yet potentially detrimental procedures such as cryopreservation. However, the mechanisms for HHP are poorly understood. We treated bovine blastocysts with three different HHP (40, 60 and 80 MPa) in combination with three recovery periods (0, 1 h, 2 h post HHP). Re-expansion rates were significantly higher at 40 and 60 but lower at 80 MPa after vitrification-warming in the treated groups than controls. Microarray analysis revealed 399 differentially expressed transcripts, representing 254 unique genes, among different groups. Gene ontology analysis indicated that HHP at 40 and 60 MPa promoted embryo competence through down-regulation of genes in cell death and apoptosis, and up-regulation of genes in RNA processing, cellular growth and proliferation. In contrast, 80 MPa up-regulated genes in apoptosis, and down-regulated protein folding and cell cycle-related genes. Moreover, gene expression was also influenced by the length of the recovery time after HHP. The significantly over-represented categories were apoptosis and cell death in the 1 h group, and protein folding, response to unfolded protein and cell cycle in the 2 h group compared to 0 h. Taken together, HHP promotes competence of vitrified bovine blastocysts through modest transcriptional changes.

*In vitro* maturation, culture and cryopreservation of gametes and embryos require meticulously adjusted conditions to avoid or minimize detrimental stress of osmotic, oxidative, cold/heat shock, nutritional and mechanical nature. Eustress can improve protein conformation, maintain cellular homeostasis as well as stabilize membrane structures, while distress will cause apoptotic cell death^1^,^2^,^3^,^4^,^5^,^6^. Recent studies indicate that stress from a well-defined and properly applied sublethal high hydrostatic pressure (HHP) may induce general adaptation and increase tolerance of various *in vitro* procedures^7^. For example, HHP treatment has been shown to improve the survival rates, fertilizing ability and development competence of cryopreserved oocytes^8^,^9^, sperm^10^,^11^, embryos^12^,^13^,^14^ and embryonic stem cells^15^. Applying a HHP of 60 MPa for 1 hour (h) increased the ICM cell numbers of bovine blastocysts^14^. Furthermore, re-expansion and hatching rates after vitrification-warming were also affected by the duration of the recovery time, i.e., the period between the termination of HHP treatment and the initiation of vitrification^13^. HHP followed by a 1 h recovery period proved to be superior with regards to both re-expansion and hatching rates^13^,^16^. Upon further fine-tuning, HHP treatment can be applied to improve *in vitro* embryo biotechnologies in various species, including humans.

The enhanced stress tolerance induced by HHP is believed to be due to differential gene expression^12^,^17^,^18^. Previous studies on mouse embryos evaluated candidate genes from pressure-related functional groups. The expression of the antizyme inhibitor 1 (*AZIN1*), the mitochondrial superoxide dismutase 2 (*SOD2*) and the gamma growth arrest and DNA-damage-inducible (*GADD45G*) were found to be significantly up-regulated by HHP treatment^12^. Further investigation revealed that pressure changed protein structures^19^,^20^ and enhanced the production of heat shock proteins (HSP), such as HSP70^14^,^19^,^20^. In bovine blastocysts subjected to HHP treatment, several candidate stress genes have also been examined. These include stress related genes *SOD2*, glutathione peroxidase 4 (*GPX4*) and heat shock 70 kDa protein 1 A (*HSPA1A*). Cumulative analysis of these genes revealed a similar pattern of expression, with a tendency for peak transcript abundance 1 h after HHP treatment^14^. This information is insufficient for explaining the molecular mode of action of the beneficial effects of HHP, therefore, the molecular mechanisms have yet to be elucidated.

Without many known definitive candidate genes, genome-wide expression profiling by DNA microarray is highly effective for the high-throughput examination of transcriptomes. Accordingly, the aim of the present study was to evaluate the effects of HHP treatments at three different levels with two different recovery times on the gene expression of bovine *in vitro* produced (IVP) vitrified blastocysts. To our knowledge, this is the first report of transcriptional profiling of bovine blastocysts treated by HHP. Pathways such as apoptosis, protein folding, cell cycle regulation, RNA processing and translation were found to be affected by HHP.

## Results

### The Effect of HHP on Re-expansion Rates of Cryopreserved Bovine IVP Blastocysts

**Figure 1:**
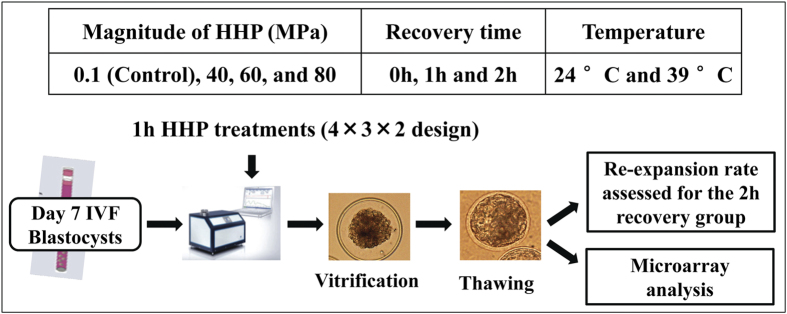
Experiment design of high hydrostatic pressure (HHP) treatment on cryopreserved bovine IVP blastocysts.

**Figure 2:**
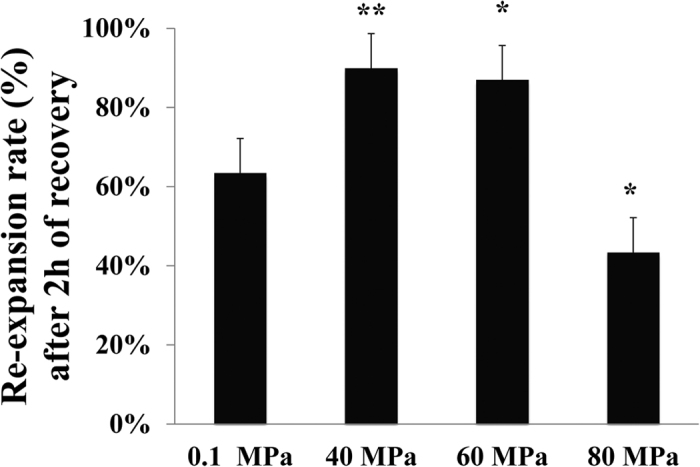
Re-expansion rates (mean ± SD) of vitrified and thawed bovine IVP blastocysts upon different HHP treatments with a 2 h-recovery time. (**P-value < 0.01, *P-value < 0.05; n = 3).

Blastocyst re-expansion is a predictive metric for implantation of frozen-thawed blastocysts. We first investigated the post-thaw survival of vitrified IVP bovine blastocysts. Because the re-expansion rate of blastocysts after 0 and 1 h recovery time had been studied previously^13^, only 2 h recovery was assessed (Fig. 1). Re-expansion rates were significantly affected by HHP and recovery time, but not by temperature (data not shown). Therefore data for the two temperatures (24 °C and 39 °C) were combined. Application of appropriate HHP (40 or 60 MPa) followed by 2 h of recovery proved to be superior with regard to re-expansion. Specifically, re-expansion rates were significantly (P < 0.05) higher in the 40-MPa (90% ± 4.5%) and 60-MPa (87% ± 3.3%) groups compared with the controls (63.5% ± 4.5%) (Fig. 2). These data demonstrated that application of defined sublethal HHP stress promoted the *in vitro* developmental competence of the vitrified bovine embryos. However, HHP treatment at 80 MPa resulted in significantly reduced re-expansion rates (43.5% ± 4.0%) compared to controls (63.5% ± 4.5%; Fig. 2), suggesting that 80 MPa was detrimental to the bovine blastocysts.

### Hierarnchical Clustering of Expression Profiles of Pressure-Treated and Cryopreserved Bovine IVP blastocysts

**Figure 3:**
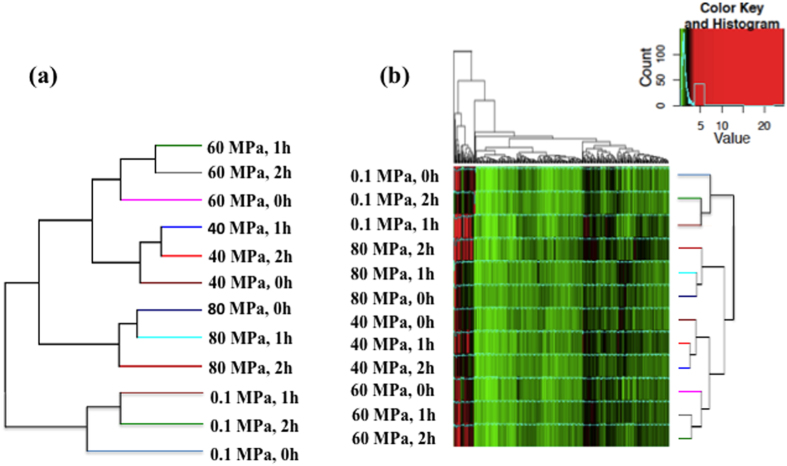
(**a**) Hierarchical clustering of all 12,274 analyzed transcripts among different HHP treatments and recovery times. The three replicates per treatments are averaged, i.e., it is a clustering by treatment and not sample. Clear separations by HHP levels (0.1, 40, 60 and 80 MPa) were seen, demonstrating that pressure is a more significant factor than recovery time. (**b**) Heatmap of differentially expressed genes among different HHP treatments. The color spectrum, ranging from red to green, indicates normalized levels of gene expression from high to low.

Building upon the previous notion and our observation that vitrified embryos re-expanded better when appropriate HHP treatment is combined with a short recovery period, a comprehensive genome-wide transcriptomic investigation was conducted. All microarray data from this study have been submitted to NCBI under the accession number of GSE7559. Hierarchical clustering of all treatments (expression data were combined for the two temperature treatments) based on 12,274 analyzed transcripts, clearly showed an effect of pressure and recovery time on gene expression profiles (Fig. 3a). Specifically, the 40- and 60-MPa groups were separated from the 80-MPa and control groups (Fig. 3a). Within each HHP level, 1 h and 2 h clustered together separate from 0 h with the exception in 80 MPa group (Fig. 3a). This overall gene expression clustering pattern was consistent with the re-expansion results.

A total of 399 transcripts (254 unique genes) were identified as differentially expressed among the different treatment groups (P < 0.05) (Supplementary Table S1). The hierarchical clustering of all differentials (Fig. 3b) was most influenced by pressure, further suggesting that pressure played a significant role in the gene expression changes.

### Effects of HHP on Gene Expression

**Figure 4:**
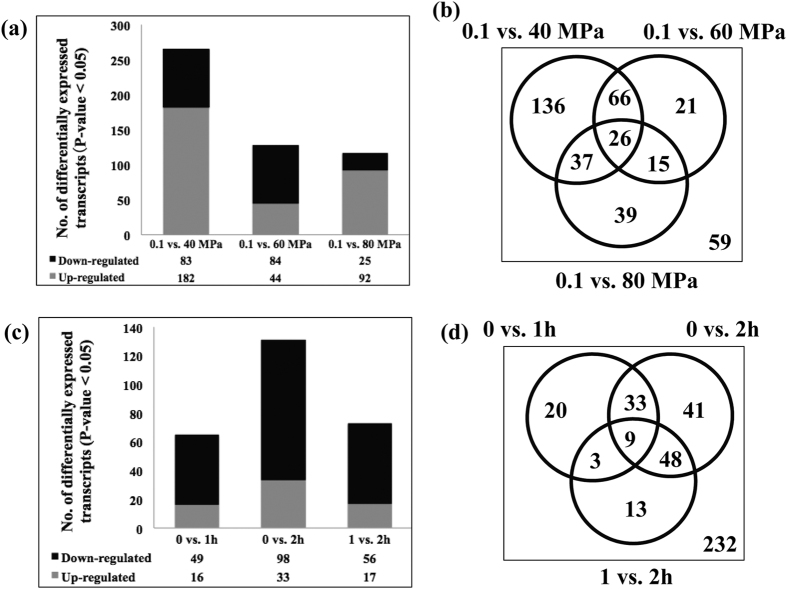
(**a**) The numbers of differentially expressed genes between HHP-treated embryos and controls. (**b**) Venn diagram shows the number of differentials specific to each comparison. (**c**) The numbers of differentially expressed genes between embryos allowed recovery time and controls. (**d**) Venn diagram shows the number of differentials specific to each comparison.

**Figure 5:**
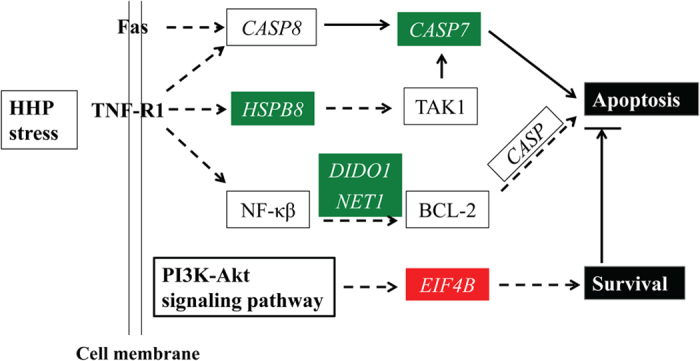
Modified apoptotic pathways in HHP-treated embryos. Genes in the green and red boxes were down- and up-regulated in both the 40- and 60-MPa treated groups (P-value < 0.05), respectively.

Among the 399 total differentially expressed transcripts, 340 were caused by HHP while 59 were not related to pressure changes. Of the 340, 83 and 182, 84 and 44, were down- and up-regulated in cryopreserved embryos treated with 40 MPa or 60 MPa HHP, respectively (Fig. 4a, Table S2). The common down-regulated transcripts in both the 40 MPa and 60 MPa HHP-treated embryos were involved in the cell death and/or apoptosis (Table 1), among these were heat shock 22 kDa protein 8 (*HSPB8*), death inducer-obliterator 1 (*DIDO1*), neuroepithelial cell-transforming gene 1 (*NET1*), coagulation factor III (*F3*) and caspase 7 (*CASP7*). Interestingly, *CASP7*, a protein of the caspase family and considered to be an important executioner protein of apoptosis, and *HSPB8*, a common heat shock protein involved in regulation of cell proliferation and apoptosis, were both down-regulated upon HHP treatment (Fig. 5). Moreover, *DIDO1* and *NET1*, both activated early in apoptosis (pro-apoptotic) were also down-regulated by HHP (Fig. 5). The expression changes of these genes upon 40 and 60 MPa HHP treatment are supportive of their potential role in the higher survival rates of these groups. Conversely, the up-regulated transcripts, such as serine/arginine-rich splicing factor 7 (*SFRS7*)*, SFRS9*, DNA-directed RNA polymerase II subunit G (*POLR2G*)*, POLR2F, POLR2L*, small nuclear ribonucleoprotein D3 (*SNRPD3*)*, SNRPD2*, eukaryotic translation initiation factor 4B (*EIF4B*), ribosomal protein L38 (*RPL38*) and mitochondrial ribosomal protein L43 (*MRPL43*) (Table 1), are involved in RNA processing and transcription, as well as regulation of protein synthesis, and likely promoted embryo survival. Collectively, these results suggested that stress caused by elevated HHP induced the embryos to degrade apoptotic transcripts and increase RNA transcription and translation. These effects, while not necessarily specific for pressure resistance or cryo-tolerance, potentially allowed the embryos to resist insults and survive more robustly. However, when pressure was further increased to 80 MPa, 25 and 92 transcripts were down- and up-regulated, respectively (Fig. 4a, Supplementary Table S2). The biological processes significantly represented among down-regulated transcripts included protein folding and cell cycle, which including BAG family molecular chaperone regulator 4 (*BAG4*), DBF4 zinc finger A (*DBF4*), mitogen-activated protein kinase phosphatase 1 (*DUSP1*), and serine/threonine-protein kinase SNK (*PLK2;* Table 1). Whereas, the up-regulated transcripts significantly over-represented were cell death, apoptosis, and chromatin assembly/disassembly, and included genes such as *EIF4B*, hexokinase 1 (*HK1*), histone cluster 1, H1e (*HIST1H1E*) and histone deacetylase 8 (*HDAC8*; Table 1). Some of these changes are opposite to those seen at 40 or 60 MPa. These results suggest that high HHP disturbs cell structure and proliferation and could be detrimental to bovine embryo survival.

Among genes up- and down-regulated at each pressure level, 136, 21 and 39 transcripts were uniquely differentially expressed in the 40-, 60- or 80-MPa treated groups compared to the controls, respectively (Fig. 4b, Supplementary Table S3). Additionally, 66 transcripts overlapped in the comparisons between the 40- or 60-MPa treated embryos and controls (Fig. 4b, Supplementary Table S4). These include down-regulated transcripts, *CASP7* and *NET1*, which is involved in cell death and apoptosis, and up-regulated transcripts, *SFRS7, SFRS9, POLR2G, POLR2F, POLR2L, SNRPD2* and amyloid beta (A4) precursor-like protein 1 (*APLP1*), which are involved in RNA processing and translation. Twenty-six transcripts were differentially expressed in all pressure treated groups compared to un-pressured controls. These include *DIDO1, HSPH1, HSPB8, HK1* and *EIF4B* (Fig. 4b, Supplementary Table S5) and may represent essential genes for pressure stress response.

### Effects of Different Recovery Time on Gene Expression

The duration of the recovery period is particularly important because HHP induces gene changes in cellular metabolism and functions and time is needed for the synthesis of related RNA and proteins. In a previous study, allowing bovine embryos to recover for 1 h after HHP increased embryo survival compared to 2 h or HHP alone without recovery^13^. Here, we compared gene expression at these three time points. Among the total of 399 differentially expressed transcripts, 167 were caused by recovery duration. We identified 49 and 98 down-regulated, and 16 and 33 up-regulated transcripts at 1 h and 2 h compared to controls (0 h), respectively (Fig. 4c, Supplementary Table S6). Gene ontology analysis of the down-regulated transcripts revealed apoptosis, proteolysis and phosphate metabolic process in the 1 h group, and protein folding, cell cycle and cell death in the 2 h group as significantly overrepresented (Table 2). Up-regulated transcripts were involved in cellular growth and proliferation, cell morphology, and cellular function and maintenance in the 1 h group, cellular growth and proliferation, DNA replication and G1/S transition of mitotic cell cycle in the 2 h group (Table 2). Of special interest was the dramatically higher number of differentially expressed transcripts in the 0 vs. 2 h comparison than that of the 0 vs. 1 h comparison, suggesting that 2 h of recovery allowed more gene expression changes to occur. Among the differentially expressed genes unique to the 0 vs. 1 h and 0 vs. 2 h comparisons, 20 and 41 transcripts were identified, respectively (Fig. 4d, Supplementary Table S7). A total of 42 transcripts were common to both the 0 vs. 1 h and 0 vs. 2 h comparisons (Fig. 4d, Supplementary Table S8), including *CASP7, DUSP1* and *F3*, which are involved in apoptosis and cell death.

It is noteworthy that although a 2 h recovery induced more gene expression changes, it did not promote better embryo re-expansion than 1 h. The additional changes in gene expression during the second hour of recovery may have corrected changes already taken place during the first hour, thereby canceling some of the changes needed to resist insults from the subsequent cryopreservation.

### Confirmation of Microarray Data by Real Time qRT-PCR

**Figure 6:**
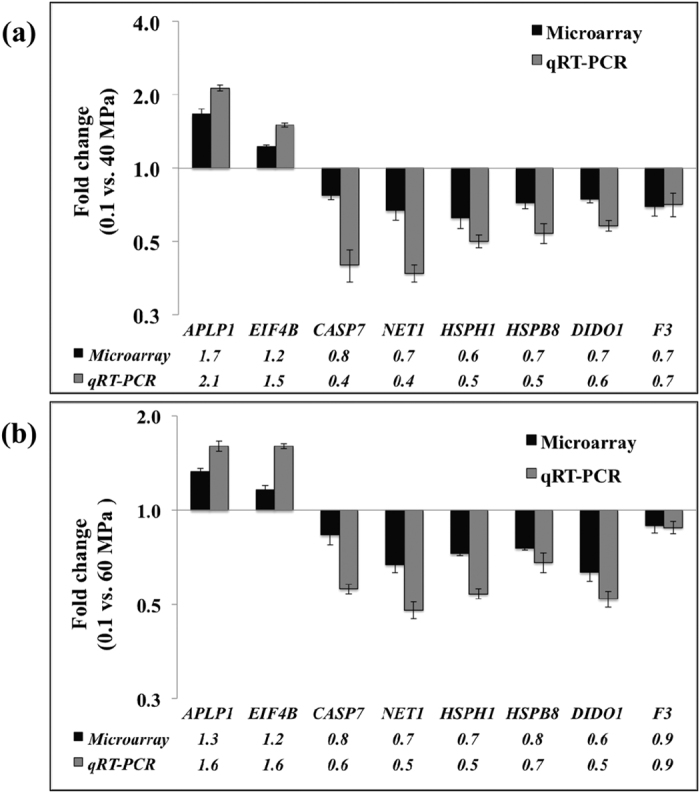
Comparisons of microarray and quantitative real-time RT-PCR (qRT-PCR) results of 8 selected genes from 40 (**a**) and 60 MPa (**b**) treated embryos. Fold change (mean ± SD) was expressed as the ratios of the levels in the 40 or 60 MPa treated embryos (n = 3) to those in the controls (0.1 MPa) (n = 3). In all cases, real time RT-PCR results substantiated the differential gene expression patterns from microarray.

To confirm the results of microarray, we performed qRT-PCR on eight genes, namely *CASP7, NET1, APLP1, EIF4B, HSPH1, HSPB8, DIDO1* and *F3*, which were significantly affected by HHP treatments and play crucial roles in cell death and apoptosis. In nearly all cases, the qRT-PCR detected greater fold changes and substantiated results of the microarray analysis (Fig. 6a,b).

## Discussion

In the bovine embryo transfer industry, vitrification is the most common method to cryopreserve IVP embryos^21^. Sublethal HHP was reported to enhance stress tolerance and increase post-thaw survival of sperm, embryos or stem cells after cryopreservation in murine, porcine and bovine^12^. Different HHP conditions (pressure level, recovery time and temperature) have been explored on gametes and embryos subjected to various assisted reproductive technologies^22^. HHP treatment at 60 MPa for 1 h has been shown to increase the *in vitro* development of bovine blastocysts^13^,^14^. In the present study, we extended previous findings by testing 40 and 80 MPa and found that 80 MPa was not well-tolerated. Additionally, we also found that HHP at 40 MPa prior to vitrification resulted in a higher re-expansion rate than the previously tested 60 MPa and the non-treated control group. The results in cattle are also in accordance with previous reports in mice and sheep^15^.

Despite advances in morphological studies, limited information on the molecular mechanisms behind the positive effect of HHP are available. Without clear candidates, comparing the transcriptomes of the treated and control embryos was the approach of choice. Interestingly, transcriptomic changes were reflective of the re-expansion data, specifically that the best treatment condition, 40 MPa, elicited the most changes in gene expression compared to controls. Most of the down-regulated genes found in the beneficial treatment levels of HHP, 40 and 60 MPa, belonged to cell death and apoptosis, while up-regulated genes were involved in RNA processing, cellular growth and proliferation, however, some of these changed in the opposite direction by the harmful level of 80 MPa HHP. The majority of the genes reported here are newly identified in HHP-treated, vitrified embryos. Collectively, it appears that while the embryos responded to HHP stress by changing gene expression, these changes prepared them for the upcoming insult of vitrification. However, when too much stress was applied (80 MPa for bovine blastocysts), embryo lethality occurred. By the candidate approach it was previously reported that sublethal stress such as heat, affects the embryos through apoptosis^2^,^3^,^23^ by influencing the expression of development^24^ and stress-related genes^25^,^26^. In this study, the same pathways were revealed for HHP stress. It appears that the embryos have limited pathways available to resist stress and use the same mechanisms for different external insults.

In accordance with the finding that a short recovery period after HHP was shown to be beneficial for cell/embryo survival^8^,^13^,^27^, we identified genes that were affected by different lengths of recovery. Similar to the effects of pressure treatment, genes involved in regulation of cell death, apoptosis and protein folding were down-regulated, while up-regulated genes belonged to cell morphology, DNA replication and cellular growth and proliferation categories after either a 1 h or 2 h recovery period. HHP and recovery seem to affect the same developmental pathways because avoiding cell death is essential for embryonic development.

In addition to the well-known apoptotic events induced by stress, we also identified many new pathways involved in potential protective mechanisms. These include RNA processing, translation, cell cycle, oxidative phosphorylation and cellular growth and proliferation. A closer look at the gene lists revealed members of the above-mentioned pathways, such as *CASP7, DIDO1, NET1, HSPH1*, and *HSPB8*, which may be responsible for possible protective mechanisms induced by HHP. In particular, *CASP7*, a member of caspase family, *HSPH1* and *HSPB8*, members from the heat shock gene family, and *DIDO1* and *NET1* were down-regulated by HHP (40 and 60 MPa). CASP7 has been shown to be an important executioner protein of apoptosis^28^,^29^. HSPH1 prevents the aggregation of denatured proteins in cells under severe stress^30^,^31^. HSPB8 belongs to the superfamily of small heat-shock proteins and is involved in regulation of cell proliferation and apoptosis through the activation of transforming growth factor-β activated kinase 1 (*TAK1*)^32^. In addition, *DIDO1* and *NET1* are activated early in apoptosis through regulation of BCL-2^33^,^34^. Sequential activation of these essential genes in response to pressure stress could play a central role in improving the stress tolerance of vitrified bovine embryos. Furthermore, *EIF4B* was up-regulated in HHP treated bovine blastocysts. *EIF4B* is required for cell proliferation and survival through regulation of protein synthesis^35^,^36^,^37^. Its up-regulation suggests that 40- and 60-MPa requires the synthesis of new proteins to promote embryo survival. However, the well-known *HSP70* (*HSPA1A* on our microarray) which was induced by sublethal pressures in microorganisms^38^ was not significantly regulated by HHP in our study. This observation is consistent with a previous report employing the candidate approach^13^. It is possible that, instead of the *HSP70*, the bovine embryos activate alternative proteins such as *HSPH1* and *HSPB8*, to respond to pressure stress.

A recent study of the transcriptome of porcine oocytes after HHP treatment revealed 44 HHP-responsive genes related to developmental process and genomic imprinting^18^. In our study, the most represented biological processes showed that apoptosis events were down-regulated while RNA processing, cellular growth and proliferation were up-regulated by 40 and 60 MPa, and a short recovery time. HHP and recovery time seem to help embryos avoid cell death and accelerate cell growth, which are essential for embryonic development. However, translation and chromatin assembly associated genes were up-regulated by appropriate HHP, which suggests that embryos go back to normal faster after warming/thawing through the assembly of important functional proteins. Interestingly, another recent study of the transcriptome of 4-cell embryos derived from HHP-treated mice oocytes showed a massive down-regulation of translation-related genes^17^. It seems that HHP puts the oocytes into energy saving mode by reducing protein synthesis. A possible reason for this discrepancy could be the difference in the dynamics of transcription across embryo development and the timing of genome activation in these three species. Also, the beneficial genes are transcribed in response to stress in a very short period in our study.

This is the first high-throughput study on HHP-treated and cryopreserved blastocysts. It should be noted that our microarray represents 10,991 genes, about half of the bovine expressed genome^39^. Of the approximately 12,000 genes expressed by the bovine blastocysts^39^, 6,086 were represented on the microarray used. Therefore, half of the blastocyst’s transcriptome is not studied here. However, we were able to identify multiple HHP-induced pathways and gene ontology categories and some that were commonly used by cells to resist other stresses, such as heat. These data suggest that the cells are limited to only a number of pathways to counteract external stress and it is therefore likely that this study identified all pathways, albeit not all genes, involved in HHP stress resistance. Application of the more powerful RNA-seq technology may help to identify additional differentially expressed genes, but it is unlikely that additional pathways will be revealed.

Previous studies mainly focused on the development of embryos from HHP-treated oocytes as well as the gene expression in treated oocytes^7^,^8^,^9^,^12^,^13^,^14^,^27^. These studies together with ours revealed that only a moderate number of genes were changed by HHP. Changes at the protein level such as folding, post-translation modifications^19^ and protein levels likely represent major responses induced by HHP. To date, proteomic analysis has only been applied in the analysis of HHP-treated microorganisms^40^. Such a study with embryos, however, is currently unfeasible due to the small number of cells that can be isolated from embryos and the high expense associated with proteomic analysis.

In summary, our results showed: 1) bovine cryopreserved embryos exhibit higher developmental competance after treatment of HHP at 40 or 60 Mpa, however, 80 MPa is not well-tolerated; 2) HHP treatments induced modest transcriptional changes in bovine embryos; and 3) HHP affected the expression of genes involved in cell death and survival, RNA processing, as well as cell cycle and cell proliferation.

## Materials and Methods

### Microarray Design and Annotation

The Cattle Array-Ready oligonucleotides were designed at the University of Illinois, Urbana-Champaign and described in detail by Everts *et al*.^41^. The microarray contained 13,254 70-mer oligonucleotide probes that were synthesized at Illumina (www.illumina.com, San Diego, CA, USA). All probes were printed in duplicate on glass slides at Microarrays Inc. (Nashville, TN, USA). In total, these oligonucleotide probes represent 10,991 unique genes.

### Collection of IVP blastocysts

*In vitro* bovine blastocysts were produced as described previously^13^,^42^. Briefly, *in vitro* fertilization (Day 0) was performed using abattoir bovine oocytes and embryos were immediately placed in CR1aa medium supplemented with BSA for Days 1 and 2 of culture. Cleaved embryos were transferred to CR1aa + 10% FBS and cultured at 38.5 °C in 5% CO_2_ in humidified air until Day 7/expanded blastocyst stage. Embryos were examined and staged under light microscopy and only morphologically intact embryos meeting the standards of Grade 1 by the International Embryo Transfer Society (IETS) were used in the following experiments.

### High Hydrostatic Pressure Treatment of Bovine IVP Blastocysts

Blastocysts were randomly distributed into the control and HHP treated groups as shown in Fig. 1. To apply HHP, groups of embryos were transferred to 0.25 ml straws in embryo holding medium (TCM-199; Gibco, Grand Island, NY, USA) without air bubbles. Straws were sealed with plastic plugs and were then placed into a pre-warmed stainless steel pressure machine (HHP machine 100; Applied Cell Technology Ltd., Hungary) with distilled water as the pressure medium. The following treatments were included (Fig. 1): (1) Control embryos were left untreated in the incubator (one atmospheric pressure or 0.1 MPa); (2) treatment groups were assigned to 40, 60 and 80 MPa HHP for 1 h at either 24 °C (room temperature) or 39 °C (body temperature), followed by three different recovery time periods (0, 1 and 2 h) post-HHP in the holding medium. The embryos were then vitrified using the Solid Surface Vitrification (SSV) method^43^. The cryopreserved blastocysts were then thawed by immersing the straws into 0.5 M sucrose solution for 5 min at 39 °C, after which the blastocysts were transferred into TCM-199 medium and cultured in an incubator at 39 °C, 5% CO_2_ and humidified air. For recorded re-expansion rates, 120 embryos were used. The re-expansion rates of the embryos were assessed after 2 h of recovery and morphological survival was determined 24 h after warming. For gene expression analysis, a total of 360 embryos were examined; pools of 5 vitrified embryos from each treatment were washed twice in D-PBS and stored in RNAlater (Ambion, Grand Island, NY, USA) in liquid nitrogen. All treatments were repeated three times (n = 3).

The re-expansion rates were analyzed using One Way ANOVA with Tukey’s HSD test. A P-value < 0.05 was considered statistically significant.

### RNA Isolation, Linear Amplification, Labeling and Microarray Hybridization

Following the reproducible procedures of RNA extraction and linear amplification from our previous study^42^, we isolated total RNA from each pool of 5 blastocysts using TRIzol reagent (Invitrogen, Grand Island, NY) and linear acrylamide as a carrier (Ambion, Grand Island, NY). The quality of the total RNA was examined with the Aglient RNA 6000 Pico kit (Aglient Technologies, Santa Clara, CA) using the Aglient Bioanalyzer 2100. The mRNA underwent two rounds of amplification using the TargetAmp 2-round aminoallyl-aRNA amplification kit 1.0 (Epicentre, Madison, WI) according to the manufacturer’s instructions. From 5 blastocysts, we were able to generate an average of 60 μg of amplified RNA. Amplified RNA was stored at −80 °C until utilization on the microarray.

The reference microarray design, in which the embryonic expression profiles were compared to a standard reference RNA, was used. The reference RNA was isolated from brain, kidney, liver and lung tissues of a naturally reproduced heifer and pooled in equal proportion. More than 90% of the probes on the microarray were hybridized by the standard reference. Two micrograms of amplified RNA from each sample and the reference were reverse transcribed, labeled, and hybridized to each microarray as previously described for single embryos^42^. In total, 144 microarrays were used including dye-swap hybridizations.

### Microarray Data Analysis

The microarrays were scanned with GenePix 400B (Molecular Devices, Union City, CA, USA) and normalization of fluorescence intensities was accomplished by using the GenePix Pro 6.0 scanning software (Axon Instruments, Union City, CA, USA). Each scanned image was examined thoroughly and dust particles and spots with high background were flagged and removed from analysis. The background and standard deviation were calculated for each raw data file after scanning, and only those spots with intensities three standard deviations above background were considered “expressed” and loaded into Genespring 12.1 (Agilent Technologies Palo Alto, CA, USA). Loess normalization was applied to all microarrays before statistical comparisons. In the analysis, each probe was considered individually.

In the post-normalization evaluation of the probes on the microarrays, 12,274 probes present in either the standard reference or the sample on 90% of the microarrays underwent further analysis. We wished to quantify the effect of temperature, recovery time, and HHP on the gene intensities. As these factors are all categorical, an ANOVA model was the natural choice. Considering a combination of the nature of the experiment and the biological focus, we decided to omit one variable from consideration to simplify the analysis. A separate ANOVA model was fit each of the possible covariates: HHP, recovery time and temperature. We looked at two metrics, firstly the number of genes for which we found that factor significant using a significance level of 0.01 and secondly the sum of the P-values for all probes. By taking into consideration both significant factors and that the re-expansion rates were not significantly affected by temperature, we chose to combine the data from the two temperatures to increase the statistical power and allow for a comprehensive analysis. We fit an ANOVA model with the covariates of HHP and recovery time. The ANOVA model returned a single P-value per probe. In order to account for multiple comparisons, we used the Benjamini Hochberg procedure to control for a false discovery rate (FDR) of 0.05. Hierarchical clusters were generated using Genespring GX 12.1 with the K-means clustering algorithm. Heatmaps and Venn diagrams of differentially expressed genes were developed with R.

### Gene Ontology Analysis

Functional annotation enrichment analysis for Gene Ontology (GO) was conducted using DAVID^44^. GO terms shown in this study summarized all similar sub-terms into an overarching term, and Benjamani-Hochberg adjusted P-values are shown for the representative term.

### Quantitative Real Time-Reverse Transcription Polymerase Chain Reaction (qRT-PCR) Analysis

qRT-PCR was performed to validate differential expression of eight selected genes using amplified RNA that used for microarray hybridization. Amplified RNA was reverse transcribed to cDNA with SuperScript III Reverse Transcriptase (Invitrogen) and amplified with specific primers designed by using Primer 3.0 (Supplementary Table S9). The qRT-PCR was performed using SYBR Green PCR Master Mix (ABI) and an ABI 7500 Fast instrument. The data were analyzed using the 7500 software version 2.0.2 provided with the instrument. All values were normalized to the internal control, *β-ACTIN*. The efficiency of each primer pair was calculated over a 3.5 log dilution range and the relative gene expression values were calculated using the 2^−ΔΔCt^ method. The same standard reference amplified RNA used in the microarray analysis was used as the calibrator sample. Expression levels that were relative to those in the standard reference were calculated and the mean for each group was determined and compared for an overall fold change. Data from qRT-PCR were analyzed as described above for re-expansion rates.

## Additional Information

**How to cite this article**: Jiang, Z. *et al*. Effects of High Hydrostatic Pressure on Expression Profiles of *In Vitro* Produced Vitrified Bovine Blastocysts. *Sci. Rep.* **6**, 21215; doi: 10.1038/srep21215 (2016).

## Supplementary Material

Supplementary Information

Supplementary Table S1

Supplementary Table S2

Supplementary Table S3

Supplementary Table S4

Supplementary Table S5

Supplementary Table S6

Supplementary Table S7

Supplementary Table S8

Supplementary Table S9

## Figures and Tables

**Table 1:** Representative gene ontology categories of differentially expressed genes affected by HHP treatment.

**Treatment**	**Change**	**GO terms**	**P-value**	**Genes**
0.1 vs. 40	Down-regulated	Cell death	1.27E-04	*DOCK1, HSPB8, F3, CASP7, RFFL, DIDO1, TOP2A, NET1*
		Apoptosis	2.55E-04	*DOCK1, F3, CASP7, RFFL, DIDO1, TOP2A, NET1*
		Transition metal ion transport	3.04E-02	*ATP2C1, SLC39A9*
	Up-regulated	RNA processing	1.16E-06	*POLR2G, POLR2F, POLR2L, SNRPD2, SART3, SF3B5, APLP1, SMNDC1, SFRS7, RBPMS, PPP1R8, SFRS9, U2AF1, DDX51*
		Translation	2.82E-02	*EIF4B, MRPL10, RPL15, RPL36, RPL38, MRPL43*
		Oxidative phosphorylation	1.42E-02	*NDUFA3, NDUFB10, NDUFB8, NDUFC2*
0.1 vs. 60	Down-regulated	Cell Death	5.47E-05	*BAG4, CASP7, DUSP6, F3, GADD45B, HK1, HSPB8, IGFBP3, DIDO1*
		Apoptosis	4.35E-02	*BAG4, GADD45B, DIDO1, NET1*
		Transition metal ion transport	3.04E-02	*ATP2C1, SLC39A9*
	Up-regulated	RNA processing	7.45E-06	*SFRS7, POLR2G, POLR2F, PRPF4B, POLR2L, SNRPD3, SFRS9, TFB2M, SNRPD2, APLP1*
		Transcription, DNA-dependent	6.58E-03	*POLR2G, BGLAP, POLR2F, POLR2L, TFB2M*
		Translation	4.34E-02	*EIF4B, RPL36, RPL38, MRPL43*
0.1 vs. 80	Down-regulated	Protein folding	1.53E-02	*BAG4, DNAJC10, DNAJA1, DNAJB6*
		Response to unfolded protein	1.86E-02	*HSPH1, DNAJA1, DNAJB6*
		Cell cycle	2.53E-02	*PLK2, DUSP1, DBF4, NSL1, ANXA1, BANP, RAD54B*
	Up-regulated	Chromatin assembly or disassembly	1.38E-05	*HIST1H1E, HDAC8*
		Apoptosis and Cell Death	3.38E-05	*AP2A2, EIF4B, HK1*
		Cellular Assembly and Organization	1.17E-02	*HDAC8*

**Table 2:** Representative gene ontology categories of differentially expressed genes affected by recovery time post-HHP.

**Treatment**	**Change**	**GO terms**	**P-value**	**Genes**
0 vs. 1h	Down-regulated	Apoptosis	1.27E-02	*F3, CASP7, MDM4, GADD45B, DIDO1*
		Proteolysis	2.02E-02	*WSB1, UHRF2, F3, CASP7, YOD1, CNOT4*
		Phosphate metabolic process	3.97E-02	*PRPF4B, DUSP1, GADD45B, IGFBP3, DUSP6*
	Up-regulated	Cellular growth and proliferation	2.64E-03	*DBF4*
		Cell Morphology	1.74E-03	*CNTNAP1*
		Cellular Function and Maintenance	1.39E-02	*NDUFA3*
0 vs. 2 h	Down-regulated	Protein folding	1.53E-02	*BAG4, DNAJC10, DNAJA1, DNAJB6, HSPH1*
		Cell cycle	2.53E-02	*FAM83D, UHRF2, PLK2, DUSP1, BANP, BIRC5, CABLES1*
		Cell death	3.92E-02	*BAG4, HSPB8, F3, CASP7, BIRC5, APLP1*
	Up-regulated	Cellular Growth and Proliferation	6.24E-05	*APLN*
		DNA replication	1.18E-02	*DBF4, WRNIP1, TOP2A*
		G1/S transition of mitotic cell cycle	4.86E-02	*BCAT1, DBF4*

## References

[b1] Silva J. L., Foguel D. & Royer C. A. Pressure provides new insights into protein folding, dynamics and structure. Trends Biochem Sci 26, 612–618 (2001). 11590014 10.1016/s0968-0004(01)01949-1

[b2] Paula-Lopes F. F. & Hansen P. J. Apoptosis is an adaptive response in bovine preimplantation embryos that facilitates survival after heat shock. Biochemical and biophysical research communications 295, 37–42 (2002). 12083763 10.1016/s0006-291x(02)00619-8

[b3] Paula-Lopes F. F. & Hansen P. J. Heat shock-induced apoptosis in preimplantation bovine embryos is a developmentally regulated phenomenon. Biology of reproduction 66, 1169–1177 (2002). 11906938 10.1093/biolreprod/66.4.1169

[b4] Horvath I. . Heat shock response in photosynthetic organisms: membrane and lipid connections. Progress in lipid research 51, 208–220 (2012). 22484828 10.1016/j.plipres.2012.02.002

[b5] Selye H. A syndrome produced by diverse nocuous agents. 1936. 10.1176/jnp.10.2.230a 9722327

[b6] Selye H. Stress without Distress. In *Psychopathology of Human Adaptation*. (ed. Serban G.) 137–146 (Springer: US,, 1976).

[b7] Pribenszky C., Molnar M., Cseh S. & Solti L. Improving post-thaw survival of cryopreserved mouse blastocysts by hydrostatic pressure challenge. Animal reproduction science 87, 143–150 (2005). 15885447 10.1016/j.anireprosci.2004.09.007

[b8] Du Y. . High hydrostatic pressure: a new way to improve *in vitro* developmental competence of porcine matured oocytes after vitrification. Reproduction 135, 13–17 (2008). 18159079 10.1530/REP-07-0362

[b9] Pribenszky C., Du Y., Molnar M., Harnos A. & Vajta G. Increased stress tolerance of matured pig oocytes after high hydrostatic pressure treatment. Animal reproduction science 106, 200–207 (2008). 18329829 10.1016/j.anireprosci.2008.01.016

[b10] Pribenszky C. . Stress preconditioning of boar spermatozoa: a new approach to enhance semen quality. Reproduction in domestic animals=Zuchthygiene 46 Suppl 2, 26–30 (2011). 21639865 10.1111/j.1439-0531.2011.01812.x

[b11] Huang S. Y. . Hydrostatic pressure pre-treatment affects the protein profile of boar sperm before and after freezing-thawing. Animal reproduction science 112, 136–149 (2009). 18538515 10.1016/j.anireprosci.2008.04.016

[b12] Bock I. . Stress Tolerance and Transcriptional Response in Mouse Embryos Treated with High Hydrostatic Pressure to Enhance Cryotolerance. Cryoletters 31, 401–412 (2010). 21042655

[b13] Siqueira E. . Vitrification of bovine blastocysts pretreated with sublethal hydrostatic pressure stress: evaluation of post-thaw *in vitro* development and gene expression. Reprod Fert Develop 23, 585–590 (2011). 10.1071/RD10203 21557925

[b14] Trigal B. . Cell counts and survival to vitrification of bovine *in vitro* produced blastocysts subjected to sublethal high hydrostatic pressure. Reproduction in domestic animals=Zuchthygiene 48, 200–206 (2013). 22775542 10.1111/j.1439-0531.2012.02131.x

[b15] Pribenszky C. . Stress for stress tolerance?A fundamentally new approach in mammalian embryology. Biology of reproduction 83, 690–697 (2010). 20554920 10.1095/biolreprod.110.083386

[b16] Pribenszky C., Siqueira F. E., Molnar M., Harnos A. & Rumpf R. Improved post-warming developmental competence of open pulled straw-vitrified *in vitro*-produced bovine blastocysts by sublethal hydrostatic pressure pretreatment. Reproduction, Fertility and Development 20, 125–125 (2008).

[b17] Bock I. . Controlled hydrostatic pressure stress downregulates the expression of ribosomal genes in preimplantation embryos: a possible protection mechanism? Reproduction, fertility, and development doi: 10.1071/RD14346, (2014). 25455885

[b18] Lin L. . Effects of high hydrostatic pressure on genomic expression profiling of porcine parthenogenetic activated and cloned embryos. Reproduction, fertility, and development 26, 469–484 (2014). 10.1071/RD13037 24618454

[b19] Silva J. L., Foguel D. & Royer C. A. Pressure provides new insights into protein folding, dynamics and structure. Trends Biochem Sci 26, 612–618 (2001). 11590014 10.1016/s0968-0004(01)01949-1

[b20] Wemekamp-Kamphuis H. H., Karatzas A. K., Wouters J. A. & Abee T. Enhanced Levels of Cold Shock Proteins in Listeria monocytogenes LO28 upon Exposure to Low Temperature and High Hydrostatic Pressure. Applied and Environmental Microbiology 68, 456–463 (2002). 11823178 10.1128/AEM.68.2.456-463.2002 PMC126669

[b21] Saragusty J. & Arav A. Current progress in oocyte and embryo cryopreservation by slow freezing and vitrification. Reproduction 141, 1–19 (2011). 20974741 10.1530/REP-10-0236

[b22] Pribenszky C. & Vajta G. Cells under pressure: how sublethal hydrostatic pressure stress treatment increases gametes’ and embryos’ performance. Reproduction, fertility, and development 23, 48–55 (2011). 10.1071/RD10231 21366980

[b23] Loureiro B., Brad A. M. & Hansen P. J. Heat shock and tumor necrosis factor-alpha induce apoptosis in bovine preimplantation embryos through a caspase-9-dependent mechanism. Reproduction 133, 1129–1137 (2007). 17636167 10.1530/REP-06-0307

[b24] Silva C. F. . Effects of heat stress on development, quality and survival of Bos indicus and Bos taurus embryos produced *in vitro*. Theriogenology 79, 351–357 (2013). 23154141 10.1016/j.theriogenology.2012.10.003

[b25] Fear J. M. & Hansen P. J. Developmental changes in expression of genes involved in regulation of apoptosis in the bovine preimplantation embryo. Biology of reproduction 84, 43–51 (2011). 20811013 10.1095/biolreprod.110.086249

[b26] Hansen P. J. To be or not to be–determinants of embryonic survival following heat shock. Theriogenology 68 Suppl 1, S40–48 (2007). 17467047 10.1016/j.theriogenology.2007.03.013

[b27] Du Y. . High hydrostatic pressure treatment of porcine oocytes before handmade cloning improves developmental competence and cryosurvival. Cloning and stem cells 10, 325–330 (2008). 18479211 10.1089/clo.2007.0089

[b28] Elmore S. Apoptosis: a review of programmed cell death. Toxicol Pathol 35, 495–516 (2007). 17562483 10.1080/01926230701320337 PMC2117903

[b29] Ouyang L. . Programmed cell death pathways in cancer: a review of apoptosis, autophagy and programmed necrosis. Cell Prolif 45, 487–498 (2012). 23030059 10.1111/j.1365-2184.2012.00845.x PMC6496669

[b30] Yamagishi N., Ishihara K., Saito Y. & Hatayama T. Hsp105 but not Hsp70 family proteins suppress the aggregation of heat-denatured protein in the presence of ADP. FEBS Letters 555, 390–396 (2003). 14644449 10.1016/s0014-5793(03)01292-4

[b31] Hosaka S. . Synthetic small interfering RNA targeting heat shock protein 105 induces apoptosis of various cancer cells both *in vitro* and *in vivo*. Cancer Sci 97, 623–632 (2006). 16827803 10.1111/j.1349-7006.2006.00217.x PMC11159471

[b32] Li B. . Overload of the heat-shock protein H11/HspB8 triggers melanoma cell apoptosis through activation of transforming growth factor-beta-activated kinase 1. Oncogene 26, 3521–3531 (2007). 17173073 10.1038/sj.onc.1210145 PMC2643355

[b33] Garcia-Domingo D., Ramirez D., Gonzalez de Buitrago G. & Martinez A. C. Death inducer-obliterator 1 triggers apoptosis after nuclear translocation and caspase upregulation. Molecular and cellular biology 23, 3216–3225 (2003). 12697821 10.1128/MCB.23.9.3216-3225.2003 PMC153187

[b34] Wu Y. Y. . Inhibition of hepatocellular carcinoma growth and angiogenesis by dual silencing of NET-1 and VEGF. J Mol Histol 44, 433–445 (2013). 23636606 10.1007/s10735-012-9480-5

[b35] Shahbazian D., Parsyan A., Petroulakis E., Hershey J. & Sonenberg N. eIF4B controls survival and proliferation and is regulated by proto-oncogenic signaling pathways. Cell cycle 9, 4106–4109 (2010). 20948310 10.4161/cc.9.20.13630 PMC3055195

[b36] Shahbazian D. . Control of cell survival and proliferation by mammalian eukaryotic initiation factor 4B. Molecular and cellular biology 30, 1478–1485 (2010). 20086100 10.1128/MCB.01218-09 PMC2832492

[b37] Dennis M. D., Jefferson L. S. & Kimball S. R. Role of p70S6K1-mediated phosphorylation of eIF4B and PDCD4 proteins in the regulation of protein synthesis. The Journal of biological chemistry 287, 42890–42899 (2012). 23105104 10.1074/jbc.M112.404822 PMC3522285

[b38] Aertsen A. . Heat shock protein-mediated resistance to high hydrostatic pressure in Escherichia coli. Appl Environ Microbiol 70, 2660–2666 (2004). 15128516 10.1128/AEM.70.5.2660-2666.2004 PMC404417

[b39] Jiang Z. . Transcriptional profiles of bovine *in vivo* pre-implantation development. BMC genomics 15, 756 (2014). 25185836 10.1186/1471-2164-15-756 PMC4162962

[b40] Martinez-Gomariz M., Hernaez M. L., Gutierrez D., Ximenez-Embun P. & Prestamo G. Proteomic analysis by two-dimensional differential gel electrophoresis (2D DIGE) of a high-pressure effect in Bacillus cereus. J Agric Food Chem 57, 3543–3549 (2009). 19338277 10.1021/jf803272a

[b41] Everts R. E. . A 7872 cDNA microarray and its use in bovine functional genomics. Veterinary immunology and immunopathology 105, 235–245 (2005). 15808303 10.1016/j.vetimm.2005.02.003

[b42] Smith S. L. . Global gene expression profiles reveal significant nuclear reprogramming by the blastocyst stage after cloning. Proceedings of the National Academy of Sciences of the United States of America 102, 17582–17587 (2005). 16314565 10.1073/pnas.0508952102 PMC1308920

[b43] Dinnyes A., Dai Y., Jiang S. & Yang X. High developmental rates of vitrified bovine oocytes following parthenogenetic activation, *in vitro* fertilization, and somatic cell nuclear transfer. Biology of reproduction 63, 513–518 (2000). 10906058 10.1095/biolreprod63.2.513

[b44] Huang da W., Sherman B. T. & Lempicki R. A. Systematic and integrative analysis of large gene lists using DAVID bioinformatics resources. Nature protocols 4, 44–57 (2009). 19131956 10.1038/nprot.2008.211

