# Supplementary material for: Effects of High Hydrostatic Pressure on Expression Profiles of In Vitro Produced Vitrified Bovine Blastocysts
Source: Sci Rep. 2016 Feb 17;6:21215. doi: 10.1038/srep21215 (PMC4756375; doi:10.1038/srep21215)
Supplement: Supplementary Information [file srep21215-s1.doc]

**Supplementary table legends**

**Effects of High Hydrostatic Pressure on Expression Profiles of In Vitro Produced Vitrified Bovine Blastocysts**

Zongliang Jiang^1,3^, Patrick Harrington^2^, Ming Zhang^3^, Sadie L. Marjani^1,4^, Joonghoon Park^1,5^, Lynn Kuo^2^, Csaba Pribenszky ^6*^, Xiuchun (Cindy) Tian^1*^

Supplementary Table S1. List of all differentially expressed genes between HHP-treated embryos and controls.

Supplementary Table S2. Differentially expressed genes between HHP-treated embryos and controls. Spreadsheet 1. Genes significantly changed upon 40 MPa treatment;

Spreadsheet 2. Genes significantly changed upon 60 MPa treatment;

Spreadsheet 3. Genes significantly changed upon 80 MPa treatment.

Supplementary Table S3. Unique differentially expressed genes between HHP-treated embryos and controls. Spreadsheet 1. Unique differentials between the 0.1 and 40 MPa groups;

Spreadsheet 2. Unique differentials between the 0.1 and 60 MPa groups;

Spreadsheet 3. Unique differentials between the 0.1 and 80 MPa groups.

Supplementary Table S4. Overlapped differentials in the comparisons between 40-, 60- or 80-MPa group and controls.

Supplementary Table S5. Differentially expressed genes common to all HHP-treated groups.

Supplementary Table S6. Differentially expressed genes between embryos with and without recovery. Spreadsheet 1. Genes differentially expressed between 0 and 1h of recovery;

Spreadsheet 2. Genes differentially expressed between 0 and 2h of recovery.

Supplementary Table S7. Unique differentials between embryos with and without recovery.

Spreadsheet 1. Unique differentials of the 0 vs. 1h comparison;

Spreadsheet 2. Unique differentials of the 0 vs. 2h comparison.

Supplementary Table S8: Overlapped differentials of the 0 vs. 1h and 0 vs. 2h comparisons.

Supplementary Table S9. Primers for real time qRT-PCR.
